# Impact of Blood Pressure on Allograft Function and Survival in Kidney Transplant Recipients

**DOI:** 10.3389/ti.2024.12574

**Published:** 2024-08-07

**Authors:** Hyo Jeong Kim, Kyung Won Kim, Young Su Joo, Junghwa Ryu, Hee-Yeon Jung, Kyung Hwan Jeong, Myung-Gyu Kim, Man Ki Ju, Seungyeup Han, Jong Soo Lee, Kyung Pyo Kang, Han Ro, Kyo Won Lee, Kyu Ha Huh, Myoung Soo Kim, Beom Seok Kim, Jaeseok Yang

**Affiliations:** ^1^ Division of Nephrology, Department of Internal Medicine, Gangnam Severance Hospital, Seoul, Republic of Korea; ^2^ Department of Internal Medicine, Korea University Guro Hospital, Korea University College of Medicine, Seoul, Republic of Korea; ^3^ Department of Internal Medicine, College of Medicine, Yongin Severance Hospital, Yonsei University, Yongin, Republic of Korea; ^4^ Department of Internal Medicine, Seoul Hospital, Ewha Womans University, Seoul, Republic of Korea; ^5^ Department of Internal Medicine, Kyungpook National University Hospital, Daegu, Republic of Korea; ^6^ Department of Internal Medicine, Kyung Hee University College of Medicine, Seoul, Republic of Korea; ^7^ Department of Internal Medicine, College of Medicine, Anam Hospital, Korea University, Seoul, Republic of Korea; ^8^ Department of Surgery, College of Medicine, Gangnam Severance Hospital, Yonsei University, Seoul, Republic of Korea; ^9^ Department of Internal Medicine, Dongsan Medical Center, Keimyung University, Daegu, Republic of Korea; ^10^ Department of Internal Medicine, Ulsan University Hospital, Ulsan, Republic of Korea; ^11^ Department of Internal Medicine, Research Institute of Clinical Medicine, Jeonbuk National University Medical School, Jeonju, Republic of Korea; ^12^ Department of Internal Medicine, Gil Hospital, Gachon University, Incheon, Republic of Korea; ^13^ Department of Surgery, Seoul Samsung Medical Center, Sungkyunkwan University, Seoul, Republic of Korea; ^14^ Department of Surgery, College of Medicine, Severance Hospital, Yonsei University, Seoul, Republic of Korea; ^15^ Department of Internal Medicine, College of Medicine, Severance Hospital, Yonsei University, Seoul, Republic of Korea

**Keywords:** kidney transplantation, graft outcome, blood pressure, time-varying, trajectory

## Abstract

The optimal target blood pressure for kidney transplant (KT) patients remains unclear. We included 808 KT patients from the KNOW-KT as a discovery set, and 1,294 KT patients from the KOTRY as a validation set. The main exposures were baseline systolic blood pressure (SBP) at 1 year after KT and time-varying SBP. Patients were classified into five groups: SBP <110; 110–119; 120–129; 130–139; and ≥140 mmHg. SBP trajectories were classified into decreasing, stable, and increasing groups. Primary outcome was composite kidney outcome of ≥50% decrease in eGFR or death-censored graft loss. Compared with the 110–119 mmHg group, both the lowest (adjusted hazard ratio [aHR], 2.43) and the highest SBP (aHR, 2.25) were associated with a higher risk of composite kidney outcome. In time-varying model, also the lowest (aHR, 3.02) and the highest SBP (aHR, 3.60) were associated with a higher risk. In the trajectory model, an increasing SBP trajectory was associated with a higher risk than a stable SBP trajectory (aHR, 2.26). This associations were consistent in the validation set. In conclusion, SBP ≥140 mmHg and an increasing SBP trajectory were associated with a higher risk of allograft dysfunction and failure in KT patients.

## Graphical Abstract

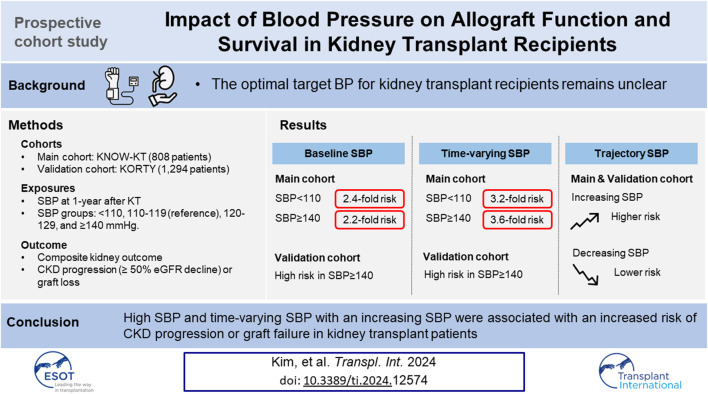

## Introduction

Post-transplant hypertension is one of the most common complications after kidney transplantation (KT). The prevalence of hypertension in kidney transplant recipients (KTRs) is reported to be approximately 50%–90% [1, 2]. Its risk factors include not only chronic kidney disease (CKD)-related risk factors, such as activation of the renin-angiotensin-aldosterone system (RAS), sympathetic nerve activity, and extracellular fluid volume expansion, but also KT-specific factors, such as calcineurin inhibitors (CNIs), corticosteroids, transplant renal artery stenosis, and angiotensin II type 1-receptor activating antibodies [3–14].

Hypertension is a well-recognized major risk factor for post-transplant cardiovascular diseases (CVD) such as congestive heart failure, ischemic heart disease, and stroke in KTRs [15–18]. Hypertension is also an independent risk factor for kidney function decline, and poor graft survival. In experimental studies, hypertension accelerates the progression of kidney failure by elevating glomerular capillary hydrostatic pressure and glomerular hyper-perfusion [19, 20]. Notably, grafted kidneys with vascular damage are likely to be susceptible to mechanical injury, which accelerates immune-mediated injury. Several clinical studies have shown the negative effect of hypertension on graft outcomes [2, 21, 22]. In an observational cohort study of living donor KTRs, the BP during the first year after KT was a significant risk factor for allograft failure, independent of kidney function [22].

Therefore, management of hypertension after KT is imperative to improve graft survival and patient survival. However, the optimal target BP for KTRs remains unclear. The SPRINT study recommended strict SBP control <120 mmHg for the reduction of cardiovascular events as well as mortality. A *post hoc* study of SPRINT also showed that intensive SBP control <120 mmHg decreased cardiovascular events in CKD patients [23, 24]. However, the ACCORD study did not find beneficial effects of strict BP control on cardiovascular events and mortality in diabetic CKD patients [25]. In the 2021 Kidney Disease Improving Global Outcomes (KDIGO) BP guidelines, the target BP for CKD patients was lowered to SBP <120 mmHg according to the SPRINT, whereas the target BP for KTRs was maintained at <130/80 mmHg [26]. No randomized controlled clinical trials (RCTs) have examined the effect of BP on CVD outcome, graft survival, or mortality in KTRs [18]. Therefore, we investigated the association between SBP and kidney outcomes in a large prospective cohort of KTRs.

## Patients and Methods

### Study Design and Participants

**FIGURE 1:**
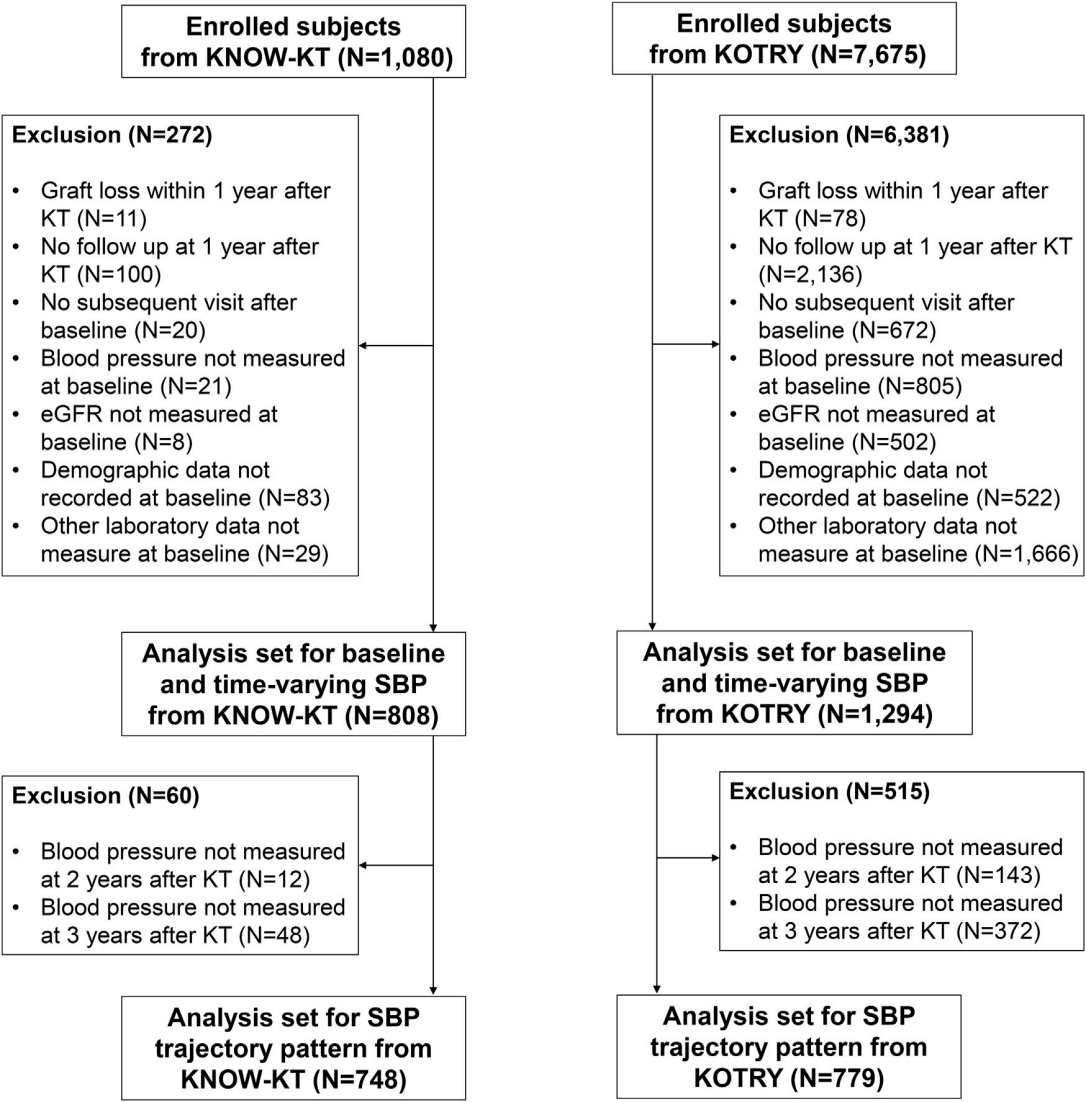
Flowchart of the enrolled study population.

The Korean Cohort Study for Outcome in Patients with Kidney Transplantation (KNOW-KT) is a multicenter, observational cohort study that investigated graft and patient outcomes along with risk factors in Korean KT patients [27]. A total of 1,080 participants were enrolled from eight Korean transplantation centers between July 2012 and August 2016 and followed up annually. We excluded 11 patients who suffered graft loss within 1 year of KT, 100 patients who had no follow-up at 1-year after KT (baseline), and 20 patients who underwent baseline examination only without subsequent visits thereafter**.** Moreover, patients with missing baseline SBP (n = 21), estimated glomerular filtration rate (eGFR) (n = 8), demographic (n = 83), and laboratory data (n = 29) were excluded. As a result, the final analysis included 808 patients (Figure 1). A total of 748 patients were enrolled in the trajectory analysis model, excluding an additional 60 patients without BP readings during the exposure period (Figure 1).

The Korean Organ Transplantation Registry (KOTRY), a nationwide cohort for organ transplantation in Korea, prospectively collected data on organ transplantation recipients and donors [28]. The KTRs between 2014 and 2020 in KOTRY were used as a validation cohort in this study. Among 7,675 eligible patients, we excluded 6,381 patients for the following reasons: graft loss within 1 year after KT (n = 78), no follow-up at 1-year after KT (n = 2,136), no subsequent visit after baseline visit (n = 672), and missing baseline SBP (n = 805), eGFR (n = 502), demographic data (n = 522), and laboratory data (n = 1,666). As a result, 1,294 patients were included in the final analysis (Figure 1).

The study was conducted in accordance with the principles of the Declaration of Helsinki and the Declaration of Istanbul, and the study protocol was approved by the Institutional Review Boards of the participating centers (4-2012-0223, 4-2014-0290). All participants provided informed consent.

### Data Collection and Measurements

Socio-demographic characteristics including age, sex, and smoking history were collected during the pre-transplant screening period. General information about transplantation, including donor-recipient relationship, recipient information including comorbidities and medications, and donor information were collected at the time of KT. The resting office BP were conducted at each yearly visit. The eGFR was calculated using the Modification of Diet in Renal Disease equation [29].

### Exposure and Outcome Ascertainment

The main exposures in this study were baseline and time-varying SBP. We defined the baseline of this study as 1 year after KT. In the time-varying analysis, we used the most recent SBP at each visit. Patients were categorized into five groups based on SBP: <110 mmHg (group 1), 110–119 mmHg (group 2, reference), 120–129 mmHg (group 3), 130–139 mmHg (group 4) and ≥140 mmHg (group 5). We performed additional analysis using the SBP trajectory determined by the differences in SBP between the baseline (1 year after KT) and after 2 years and between the baseline and after 3 years.

The primary outcome was the composite kidney outcome of CKD progression or graft loss. CKD progression was defined as a ≥50% decline in eGFR from baseline values. Graft loss was defined as the requirement for maintenance dialysis for more than 3 months or re-transplantation. Patients were censored at the date of the last visit, all events or death.

### Statistical Analysis

Continuous variables are expressed as mean values with standard deviation for normally distributed data or medians with interquartile ranges (IQRs) for skewed data. The Kolmogorov–Smirnov test was performed to determine the normality of all continuous variables. Categorical variables are presented as frequencies with percentage. Comparisons between groups were performed using a one-way analysis of variance or Kruskal–Wallis test for continuous variables, as appropriate. The chi-squared test or Fisher’s exact test was used for comparing categorical variables. Cox proportional hazards regression analysis evaluated the association between baseline SBP and study outcomes. In addition, we constructed marginal structural Cox models to reflect time-dependent changes in SBP and other covariates. In BP trajectory modeling, we used group-based trajectory modeling to categorize the trend of BP over time. The longitudinal BP was fitted as a mixture of multiple latent trajectories in a censored normal model with a polynomial function of time [30, 31]. Death events before the incidence of composite kidney outcome and loss to follow-up were treated by censoring at the date of death and the last examination, respectively. Significant variables related to CKD progression or graft loss in univariate analysis (*p* < 0.10) were included into all models for adjustment. Model 1 characterizes the crude hazard ratio (HR) without adjustment. Model 2 was adjusted for age, sex, body mass index (BMI), smoking status, diabetes mellitus (DM), CVD, eGFR, hemoglobin, albumin, low-density lipoprotein cholesterol (LDL-C), ABO compatibility, HLA compatibility, delayed graft function (DGF), acute rejection during the first year, donor type (living vs. deceased), donor age, donor BMI, donor hypertension, donor eGFR, and immunosuppressants (tacrolimus, cyclosporine, and steroids). Model 3 was further adjusted for BP-lowering medications (RAS blockers, beta-blockers, calcium channel blockers, alpha-blockers, and diuretics). In the trajectory model, baseline SBP was additionally adjusted. The results from multivariable hazard models are presented as HRs and 95% confidence intervals (CIs). All statistical analyses were performed with Stata 14 statistical software (StataCorp, College Station, TX), with a *p*-value <0.05 considered significant.

## Results

### Baseline Characteristics

Table 1 shows baseline characteristics of 808 participants according to baseline SBP categories. The mean age of participants was 45.8 ± 11.4 years, and 62.7% were women. Almost all patients (96.0%) had hypertension, and the mean SBP and DBP were 124.3 ± 12.6 and 78.7 ± 10.7 mmHg, respectively. The mean baseline eGFR was 64.7 ± 18.0 mL/min/1.73 m^2^. Numbers of patients with SBP <110, 110–119, 120–129, 130–140, and ≥140 mmHg were 93 (11.5%), 168 (20.8%), 292 (36.1%), 164 (20.3%), and 91 (11.3%), respectively. Patients with SBP ≥140 mmHg were older, more likely to be women, and had higher BMI. Moreover, those with highest SBP had more DM and treated with more RAS blockers, and beta-blockers than those with lower SBP groups.

**TABLE 1:** Baseline characteristics of participants according to systolic blood pressure categories.

	SBP category (mmHg)						
	Total	<110	110–119	120–129	130–139	≥140	*p*-value
Demographic data							
N (%)	808 (100)	93 (11.5)	168 (20.8)	292 (36.1)	164 (20.3)	91 (11.3)	
Age (years)	45.8 ± 11.4	46.5 ± 10.6	44.5 ± 11.4	44.9 ± 11.7	46.8 ± 11.2	48.3 ± 10.7	0.034
Female, n (%)	507 (62.7)	49 (52.7)	99 (58.9)	187 (64.0)	105 (64.0)	67 (73.6)	0.040
BMI (kg/m^2^)	22.6 ± 3.2	21.7 ± 3.0	22.3 ± 2.9	22.8 ± 3.4	22.9 ± 3.3	23.5 ± 3.3	0.001
SBP (mmHg)	124.3 ± 12.6	103.2 ± 4.8	114.9 ± 2.9	124.1 ± 2.8	133.7 ± 2.8	146.7 ± 6.5	<0.001
DBP (mmHg)	78.7 ± 10.7	66.3 ± 7.1	74.5 ± 7.3	78.5 ± 8.8	84.0 ± 8.3	89.7 ± 11.6	<0.001
Diabetes mellitus, n (%)	207 (26.2)	34 (37.0)	28 (17.3)	69 (24.3)	38 (23.6)	38 (41.8)	<0.001
Hypertension, n (%)	759 (96.1)	86 (93.5)	155 (95.7)	271 (95.4)	158 (98.1)	89 (97.8)	0.330
Coronary artery disease, n (%)	47 (5.9)	4 (4.3)	13 (8.0)	15 (5.3)	6 (3.7)	9 (9.9)	0.210
Cerebrovascular disease, n (%)	28 (3.5)	7 (7.6)	3 (1.9)	13 (4.6)	3 (1.9)	2 (2.2)	0.075
Congestive heart failure, n (%)	13 (1.6)	1 (1.1)	4 (2.5)	5 (1.8)	1 (0.6)	2 (2.2)	0.720
Smoker, n (%)							0.730
Never	434 (53.7)	51 (54.8)	90 (53.6)	159 (54.5)	88 (53.7)	46 (50.5)	
Current	52 (6.4)	10 (10.8)	11 (6.5)	18 (6.2)	7 (4.3)	6 (6.6)	
Former	322 (39.9)	32 (34.4)	67 (39.9)	115 (39.4)	69 (42.1)	39 (42.9)	
Donor, n (%)							0.920
Living donor	682 (84.4)	80 (86.0)	145 (86.3)	244 (83.6)	137 (83.5)	76 (83.5)	
Deceased or DCD	126 (15.6)	13 (14.0)	23 (13.7)	48 (16.4)	27 (16.5)	15 (16.5)	
Donor age (years)	45.2 ± 11.7	43.8 ± 12.5	43.6 ± 11.3	45.6 ± 11.5	45.8 ± 12.0	47.7 ± 11.6	0.052
Donor BMI (kg/m^2^)	23.8 ± 2.9	23.8 ± 2.7	23.6 ± 2.8	23.8 ± 3.1	23.7 ± 2.9	23.9 ± 2.8	0.920
Donor hypertension, n (%)	90 (37.3)	4 (21.1)	18 (35.3)	36 (37.5)	20 (41.7)	12 (44.4)	0.520
ABO-incompatibility, n (%)	147 (18.2)	21 (22.6)	29 (17.3)	55 (18.8)	21 (12.8)	21 (23.1)	0.200
Delayed graft function, n (%)	6 (0.7)	0 (0.0)	2 (1.2)	2 (0.7)	2 (1.2)	0 (0.0)	0.670
Laboratory parameters							
eGFR (ml/min/1.73 m^2^)	64.7 ± 18.0	65.9 ± 17.9	66.5 ± 18.4	65.1 ± 18.4	62.4 ± 17.3	63.0 ± 16.6	0.220
Donor eGFR (ml/min/1.73 m^2^)	99.2 ± 40.9	99.1 ± 27.3	101.9 ± 29.9	99.0 ± 53.4	96.8 ± 36.9	99.5 ± 29.7	0.870
Hemoglobin (g/dL)	13.5 ± 1.9	13.3 ± 1.8	13.5 ± 2.0	13.5 ± 1.9	13.6 ± 1.9	13.9 ± 1.8	0.380
Albumin (g/dL)	4.4 ± 0.3	4.4 ± 0.3	4.3 ± 0.4	4.4 ± 0.3	4.4 ± 0.3	4.4 ± 0.3	0.260
Fasting glucose (mg/dL)	109.9 ± 37.0	109.0 ± 31.3	104.9 ± 30.2	111.4 ± 37.1	109.8 ± 42.6	115.2 ± 42.5	0.270
T-Chol (mg/dL)	178.0 ± 36.6	173.1 ± 41.5	174.0 ± 36.3	178.5 ± 35.4	182.4 ± 36.0	181.0 ± 36.5	0.150
LDL-C (mg/dL)	96.7 ± 30.6	96.2 ± 32.8	91.9 ± 31.0	97.9 ± 30.0	97.8 ± 29.6	100.2 ± 30.6	0.200
HDL-C (mg/dL)	58.4 ± 17.4	57.9 ± 17.3	59.3 ± 18.1	58.1 ± 16.3	59.7 ± 19.0	55.7 ± 16.2	0.450
Triglyceride (mg/dL)	136.7 ± 96.0	115.5 ± 45.0	128.3 ± 85.7	142.3 ± 114.9	143.5 ± 101.6	143.7 ± 69.6	0.088
Drugs							
Tacrolimus, n (%)	755 (93.4)	88 (94.6)	155 (92.3)	275 (94.2)	154 (93.9)	83 (91.2)	0.800
Cyclosporine, n (%)	42 (5.2)	5 (5.4)	11 (6.5)	12 (4.1)	8 (4.9)	6 (6.6)	0.790
Steroid, n (%)	744 (92.1)	86 (92.5)	157 (93.5)	263 (90.1)	148 (90.2)	90 (98.9)	0.071
RAS blockers, n (%)	120 (14.9)	6 (6.5)	17 (10.1)	50 (17.1)	27 (16.5)	20 (22.0)	0.010
Diuretics, n (%)	50 (6.2)	5 (5.4)	10 (6.0)	19 (6.5)	8 (4.9)	8 (8.8)	0.790
Beta-blockers, n (%)	274 (33.9)	22 (23.7)	54 (32.1)	94 (32.2)	58 (35.4)	46 (50.5)	0.003
Calcium channel blockers, n (%)	379 (46.9)	21 (22.6)	76 (45.2)	139 (47.6)	92 (56.1)	51 (56.0)	<0.001
Alpha blockers, n (%)	15 (1.9)	2 (2.2)	2 (1.2)	5 (1.7)	4 (2.4)	2 (2.2)	0.930

Data are expressed as mean ± standard deviation, median [interquartile range], or proportion n (%).

Abbreviations: BMI, body mass index; SBP, systolic blood pressure; DBP, diastolic blood pressure; DCD, donation after circulatory death; eGFR, estimated glomerular filtration rate; T-Chol, total cholesterol; LDL-C, low-density lipoprotein cholesterol; HDL-C, high-density lipoprotein cholesterol; RAS blockers, renin-angiotensin system blockers.

### Association of SBP With Adverse Kidney Outcomes

During a median follow-up period of 5.93 years, 85 (10.5%) participants reached the primary composite outcome and the overall incidence rate was 19.3 per 1,000 person-years (Table 2). The primary composite outcome of CKD progression or graft loss occurred in 15 (16.1%), 13 (7.7%), 29 (9.9%), 15 (9.1%), and 13 (14.3%) patients in groups 1 (SBP <110 mmHg), 2 (SBP 110–119 mmHg), 3 (SBP 120–129 mmHg), 4 (SBP 130–139 mmHg), and 5 (SBP ≥140 mmHg), respectively.

**TABLE 2:** The CKD progression^a^, graft loss, and composite outcome^b^ rates according to baseline SBP.

Outcomes	SBP categories (mmHg)					
	Overall	<110	110–119	120–129	130–139	≥140
No. of participants, n (%)	808	93 (11.5)	168 (20.8)	292 (36.1)	164 (20.3)	91 (11.3)
CKD progression						
No. of person-years	4386.9	497.1	906.0	1591.6	905.0	487.2
Incidence of outcome, n (%)	76 (9.4)	13 (14.0)	9 (5.4)	26 (8.9)	15 (9.1)	13 (14.3)
Incidence rate per 1,000 person-year	17.3	26.2	9.9	16.3	16.6	26.7
Graft loss						
No. of person-years	5744.1	644.7	1188.2	2084.0	1191.1	636.1
Incidence of outcome, n (%)	36 (4.5)	8 (8.6)	6 (3.6)	10 (3.4)	7 (4.3)	5 (5.5)
Incidence rate per 1,000 person-year	6.3	12.4	5.0	4.8	5.9	7.9
Kidney composite outcome						
No. of person-years	4400.3	495.8	910.4	1602.1	904.9	487.1
Incidence of outcome, n (%)	85 (10.5)	15 (16.1)	13 (7.7)	29 (9.9)	15 (9.1)	13 (14.3)
Incidence rate per 1,000 person-year	19.3	30.3	14.3	18.1	16.6	26.7

Abbreviations: CKD, chronic kidney disease; SBP, systolic blood pressure; eGFR, estimated glomerular filtration rate.

^a^ CKD progression was defined as a decline of ≥50% in eGFR.

^b^ Composite outcome was defined as CKD progression or graft loss.

**FIGURE 2:**
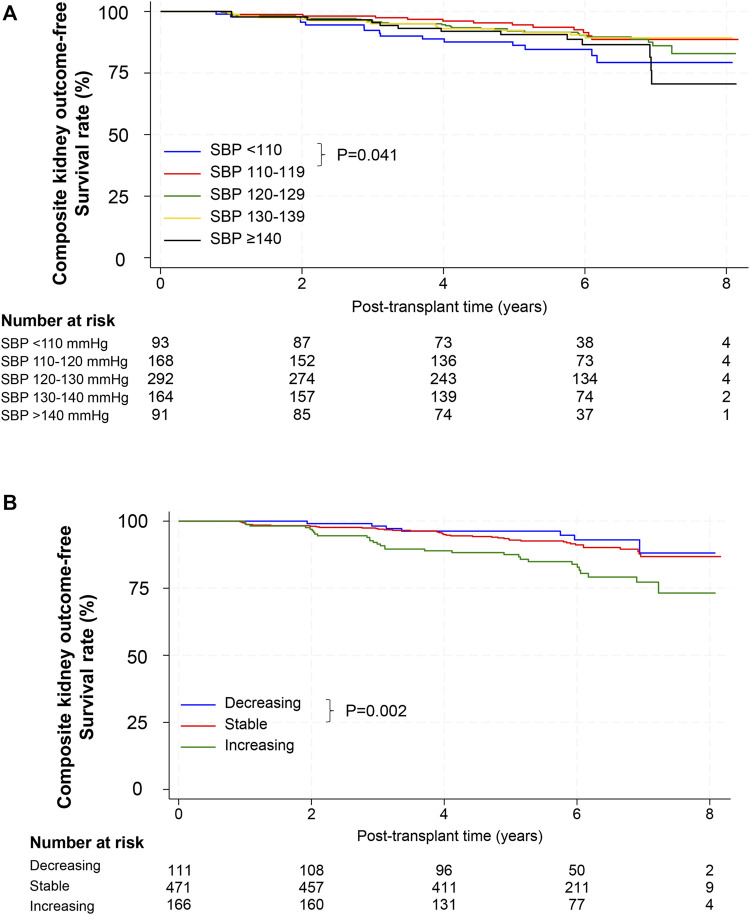
Composite outcomes according to SBP. **(A)** Kaplan–Meier survival curves for composite outcomes after kidney transplantation according to baseline SBP. *p*-value, comparison for the 110–119 mmHg group by the log-rank test. **(B)** Kaplan–Meier survival curves for composite outcomes after kidney transplantation according to SBP trajectory. *p*-value, comparison for the stable group by the log-rank test. Abbreviations: SBP, systolic blood pressure.

When the cumulative incidence of the primary composite outcomes was compared between the baseline SBP groups using the log-rank test, group 2 (SBP 110–119 mmHg) showed a lower incidence than group 1 (SBP <110 mmHg, *p* = 0.041) and a trend of lower incidence than group 5 (SBP ≥140 mmHg, *p* = 0.085) (Figure 2A). In Cox regression analysis, the risk of CKD progression or graft loss increased in both group 1 and 5 compared with that in group 2. After adjustment for potential confounding factors, the adjusted HRs for groups 1 and 5 were 2.43 (95% confidence interval [CI], 1.12–5.26) and 2.25 (1.00–5.02), respectively, compared with the reference group 2 (Table 3).

**TABLE 3:** The hazard ratios for the composite outcome of CKD progression or graft failure according to baseline SBP or time-varying SBP.

Baseline SBP	Model 1		Model 2		Model 3	
	HR (95% CI)	*p*-value	HR (95% CI)	*p*-value	HR (95% CI)	*p*-value
<110	2.13 (1.01–4.48)	0.046	2.31 (1.07–4.98)	0.032	2.43 (1.12–5.26)	0.024
110–119	1.00		1.00		1.00	
120–129	1.26 (0.65–2.42)	0.492	1.21 (0.62–2.35)	0.579	1.22 (0.63–2.39)	0.552
130–139	1.18 (0.56–2.47)	0.670	1.26 (0.59–2.70)	0.547	1.26 (0.59–2.70)	0.550
≥140	1.91 (0.88–4.12)	0.099	2.21 (0.99–4.94)	0.053	2.25 (1.00–5.02)	0.049

Time-varying SBP	Model 1		Model 2		Model 3	
	HR (95% CI)	*p*-value	HR (95% CI)	*p*-value	HR (95% CI)	*p*-value
<110	2.14 (0.85–5.40)	0.107	2.99 (1.10–8.09)	0.031	3.02 (1.11–8.22)	0.030
110–119	1.00		1.00		1.00	
120–129	1.95 (0.91–4.16)	0.085	2.30 (0.99–5.37)	0.054	2.29 (0.98–5.35)	0.055
130–139	1.68 (0.75–3.74)	0.204	2.08 (0.85–5.12)	0.109	2.06 (0.84–5.07)	0.116
≥140	3.20 (1.48–6.89	0.003	3.67 (1.52–8.82)	0.004	3.60 (1.48–8.72)	0.005

Model 1: Unadjusted. Model 2: Adjusted for age, sex, BMI, smoking status, DM, CVD, ABO compatibility, HLA compatibility, DGF, acute rejection during the first year, type of kidney donor (living or deceased donor), donor age, donor eGFR, donor BMI, donor hypertension, laboratory parameters (eGFR, hemoglobin, albumin, and LDL-C), and immunosuppressant use (tacrolimus, cyclosporine, and steroid). Model 3: Model 2 + BP-lowering drugs (RAS inhibitors, beta-blockers, calcium channel blockers, alpha-blockers, and diuretics).

Abbreviations: CKD, chronic kidney disease; SBP, systolic blood pressure; HR, hazard ratio; CI, confidence interval; BMI, body mass index; DM, diabetes mellitus; CVD, cardiovascular disease; HLA, human leukocyte antigen; DGF, delayed graft function; eGFR, estimated glomerular filtration rate; LDL-C, low-density lipoprotein cholesterol; RAS, inhibitors, renin-angiotensin system inhibitors.

Next, we examined the association of time-varying SBP levels with the composite kidney outcome using a marginal structural Cox regression model. In the fully adjusted model, group 1 and 5 had a 3.02 (95% CI 1.11–8.22) and 3.60 (95% CI, 1.48–8.72) -fold higher risk of composite outcomes than the reference group 2 (Table 3).

### Association of SBP Trends With Adverse Kidney Outcomes

We further analyzed the association between SBP trends and adverse kidney outcomes. Incidence rates of composite kidney outcomes in the decreasing, stable, and increasing SBP trajectory groups were 11.2, 15.8, and 32.8 per 1,000 person-years, respectively (Table 4). The stable SBP group showed better outcomes than the increasing SBP group by log-rank test (*p* = 0.002, Figure 2B). In a fully adjusted Cox model, the HR for the increasing SBP trajectory group was 2.26 (95% CI, 1.34–3.81) compared with the stable SBP trajectory group (Table 5). The decreasing SBP trajectory group showed better outcomes than the stable SBP trajectory group (HR, 0.63; 95% CI 0.26–1.51, Table 5); however, the difference was not statistically significant.

**TABLE 4:** Outcome event rates according to SBP trajectory pattern.

Outcomes	SBP trajectory pattern			
	Overall	Decreasing	Stable	Increasing
No. of participants n (%)	748 (100.0)	111 (14.8)	471 (63.0)	166 (22.2)
≥50% decline in eGFR^a^				
No. of person-years	4187.2	625.5	2653.2	908.5
Incidence of outcome, n (%)	72 (9.6)	7 (6.3)	37 (7.9)	28 (16.9)
Incidence rate per 1,000 person-year	17.2	11.2	13.9	30.8
Graft loss				
No. of person-years	4688.2	691.9	2972.7	1023.6
Incidence of outcome, n (%)	35 (4.7)	0 (0.0)	20 (4.2)	15 (9.0)
Incidence rate per 1,000 person-year	7.3	0	6.4	14.7
Kidney composite outcome^b^				
No. of person-years	4196.0	625.5	2656.5	914.0
Incidence of outcome, n (%)	79 (10.5)	7 (6.3)	42 (8.9)	30 (18.1)
Incidence rate per 1,000 person-year	18.8	11.2	15.8	32.8

Abbreviations: CKD, chronic kidney disease; SBP, systolic blood pressure; eGFR, estimated glomerular filtration rate.

^a^ CKD progression was defined as a decline of ≥50% in eGFR.

^b^ Composite outcome was defined as CKD progression or graft loss.

**TABLE 5:** The hazard ratios for the composite outcome of CKD progression or graft failure according to SBP Trajectory Patterns.

SBP trajectory	Model 1		Model 2		Model 3	
	HR (95% CI)	*p*-value	HR (95% CI)	*p*-value	HR (95% CI)	*p*-value
Decreasing	0.72 (0.32–1.60)	0.417	0.62 (0.26–1.49)	0.287	0.63 (0.26–1.51)	0.302
Stable	1.0		1.0		1.00	
Increasing	2.06 (1.29–3.30)	0.002	2.33 (1.38–3.92)	0.002	2.26 (1.34–3.81)	0.002

Model 1: Unadjusted. Model 2: Adjusted for baseline SBP, age, sex, BMI, smoking status, DM, CVD, ABO compatibility, HLA compatibility, DGF, acute rejection during the first year, type of kidney donor (living or deceased donor), donor age, donor eGFR, donor BMI, donor hypertension, laboratory parameters (eGFR, hemoglobin, albumin, and LDL-C), and immunosuppressant use (tacrolimus, cyclosporine, and steroid). Model 3: Model 2 + BP-lowering drugs (RAS blockers, beta-blockers, calcium channel blockers, alpha-blockers, and diuretics).

Abbreviations: CKD, chronic kidney disease; SBP, systolic blood pressure; HR, hazard ratio; CI, confidence interval; BMI, body mass index; DM, diabetes mellitus; CVD, cardiovascular disease; HLA, human leukocyte antigen; DGF, delayed graft function; eGFR, estimated glomerular filtration rate; LDL-C, low-density lipoprotein cholesterol; RAS inhibitors, renin-angiotensin system inhibitors.

### Association of SBP or SBP Trends With Adverse Kidney Outcomes in the Validation Cohort

Supplementary Table S1 shows the baseline characteristics of 1,294 participants according to baseline SBP categories. The mean age of participants was 47.8 ± 11.4 years, and 44.1% were women. The prevalence of hypertension was 75.2%, and the mean SBP and DBP were 125.4 ± 14.0 and 77.0 ± 11.0 mmHg, respectively. The mean baseline eGFR was 64.5 ± 18.3 mL/min/1.73 m^2^. The numbers of patients with SBP <110, 110–119, 120–129, 130–139, and ≥140 mmHg were 156 (12.1%), 241 (18.6%), 402 (31.1%), 308 (23.8%), and 17 (14.5%), respectively.

During a median follow-up period of 2.29 years, the overall incidence of the primary composite outcome was 17.2 per 1,000 person-years (Supplementary Table S2). The primary composite outcome occurred in 7 (4.5%), 6 (2.5%), 18 (4.5%), 13 (4.2%), and 14 (7.5%) patients in groups 1 (SBP <110 mmHg), 2 (SBP 110–119 mmHg), and 3 (SBP 120–129 mmHg), 4 (SBP 130–139 mmHg), and 5 (SBP ≥140 mmHg), respectively.

Although the risk of composite kidney outcome was high in group 5 (SBP ≥140 mmHg) than in group 2 (SBP 110–119 mmHg) of this validation cohort (HR, 3.85; 95% CI 1.42–10.43) in parallel with the discovery cohort, there was no statistically significant increase in risk in group 1 (SBP <110 mmHg) (Supplementary Table S3).

When the association of time-varying SBP levels with the composite kidney outcome was analyzed in the validation cohort, the group with group 5 had a 4.16-fold higher risk of composite kidney outcome than the reference group with group 2 similar to the discovery cohort (Supplementary Table S3).

When the cumulative incidence of the primary composite outcomes was compared between the SBP trajectory groups using multivariable Cox regression analysis, the trend was similar in the validation cohort as in the discovery cohort that the increasing SBP trajectory was associated with a higher risk of adverse kidney outcome compared with the stable SBP trajectory (HR, 2.75; 95% CI 1.10–6.84, Supplementary Table S4).

## Discussion

In this study, we examined the association of baseline and time-varying SBP after kidney transplantation with composite kidney outcomes reflecting allograft function. Furthermore, we identified three patterns of SBP trends using trajectory modeling and evaluated the association between SBP trends and adverse kidney outcomes in KTRs. We found that baseline SBP at 1 year after transplantation higher than 140 mmHg was associated with a higher risk of adverse kidney outcomes of CKD progression or graft failure. Additionally, the risk of adverse kidney outcomes was 3.60-fold higher in patients with time-varying SBP ≥140 mmHg than in those with well-controlled SBP of 110–119 mmHg. In the BP trajectory model, the increasing BP trajectory was associated with a higher risk of composite kidney outcomes than those with a stable BP trajectory. Our findings suggest that chronically elevated BP after transplantation is associated with a declining kidney function in KTRs.

Hypertension is a well-established, major cause of cardiovascular events and a non-immunological factor of graft loss for KTRs [16, 32–35]. However, no prospective RCTs have studied the association of optimal BP targets with clinically significant outcomes, including CVD, graft survival, and mortality. The latest 2021 KDIGO and 2017 ACC/AHA guidelines recommended a target of BP less than 130/80 mmHg in KTRs [26, 36]. Current guidelines are mainly based on retrospective studies and registry data. The *post hoc* analysis of the FAVORIT trial showed that higher SBP is independently associated with an increased risk of CVD and all-cause mortality in KTRs [18]. The Collaborative Transplant Study registry examined the impact of post-transplant BP on long-term kidney graft outcomes in 29,751 deceased donor KTR [32]. This study concluded that increased BP is associated with functional graft loss. A US single-center study studied the relationship between blood pressure adjusted for renal function and allograft survival in 277 deceased donor KTR [21]. They showed that elevated SBP, DBP, and mean arterial BP at 1-year post-transplantation were significantly associated with allograft survival independent of baseline renal allograft function. Several prior studies for deceased donor KTRs, have examined the association of BP and allograft survival [21, 32, 37]. For living donor KTRs, a US single center study with 392 KTRs reported that BP during the first year after transplantation is a significant factor of allograft failure independent of renal function [22].

Our discovery cohort analysis suggested a U-shaped association of SBP at 1 year after KT with an increased risk of adverse kidney graft outcomes in Korean KTRs. The denervation status of kidney allografts and CNI may impair myogenic autoregulation, leading to a higher risk for acute kidney injury and more rapid loss of kidney function with low BP [38]. In parallel, a retrospective study also suggests controlling SBP within the range of 121–130 mmHg and implies that overly strict control of SBP below 120 mmHg might impair kidney allograft function [39]. However, our validation cohort analysis failed to confirm a significantly higher risk of low SBP, although it confirmed a higher risk of high SBP. Similarly, a conflicting result have been reported in previous studies. A *post hoc* analysis of the FAVORIT trial reported that low SBP <110 mmHg was not associated with a higher risk for eGFR decline or allograft failure in KTRs with no evidence of a “U” shaped relationship [38]. Although high SBP is universally acknowledged as a risk factor [2, 18, 22, 32, 40], the optimal range of SBP to maximize graft and patient survival remains a topic of ongoing research.

In time-varying analysis and trajectory models, we showed that chronically high SBP and persistently increasing SBP have adverse effects on allograft function. Despite ongoing debate regarding the optimal SBP target, our findings underscore the critical importance of not only achieving optimal BP levels but also implementing regular monitoring and management of BP in KTRs. These results emphasize the necessity for healthcare providers to closely track and adjust treatment plans in response to fluctuations in blood pressure.

This study had several strengths, although many findings are consistent with those of prior seminal studies. First, while previous studies mainly studied the association between baseline BP at spot time and graft failure, our study investigated the association between time-varying SBP and graft outcomes. Since a time-varying analysis was performed, it was possible to reflect BP fluctuation over time, and the effect of long-term BP after transplantation on graft outcomes could be evaluated. Moreover, this study examined the temporal association of various BP trends with the risk of graft outcomes by trajectory modeling. Second, this was an intermediate-sized, multicenter transplant study with complete follow-up data collected prospectively over several years. Furthermore, we implemented the same analysis using a validated cohort of large, nationwide population to support our main findings. Third, we included recipients who received kidney grafts from living and deceased donors to reflect real-world situations and adjusted covariates related to KT-specific factors, such as donor characteristics, DGF, and compatibility of donors and recipients to minimize the influence of transplant-related factors that could affect kidney graft function. Fourth, as the first Asian data, this study can contribute to generalization of the previous results derived from the Western countries.

This study had several limitations. First, owing to the observational design of this study, our results cannot prove causality between SBP and adverse kidney outcomes, and all potential confounding factors could not be completely controlled. However, this study consisted of a large and homogeneous population, and multiple potential confounding factors were included in the adjustment model. Second, the SBP used as the baseline was based on a single measurement. To overcome this limitation, we employed time-varying and trajectory statistical method, further supporting our primary study results. Third, there was a discrepancy between the study results using the discovery and validation cohort. In the discovery cohort, although not statistically significant, the risk of adverse kidney outcomes tended to increase in the group with time-varying SBP less than 110 mmHg, whereas it seemed to decrease in the validation cohort. There were several differences between the two cohorts. Comparing the baseline characteristics of participants in the two cohorts, deceased donor KT occupied a larger proportion in the validation cohort than in the discovery cohort. The medication use could not be adjusted in the regression model since there was no information on medications, including immunosuppressants and BP-lowering medications, in the validation cohort. In addition, the validation cohort had a shorter median follow-up period than the discovery cohort. Further, large-scale, randomized, controlled trials with longer follow-up periods are needed to confirm the present results for the optimal BP target and the impact of BP on kidney allograft outcomes in KTRs.

In conclusion, high SBP (≥140 mmHg) at 1 year after KT was associated with an increased risk of CKD progression or graft failure in KTRs. A higher time-varying SBP (≥140 mmHg) and an increasing trend of SBP were also associated with an increased risk of adverse kidney allograft outcomes.

## Data Availability

The raw data supporting the conclusions of this article will be made available by the authors, without undue reservation.
