# Supplementary material for: Impact of Blood Pressure on Allograft Function and Survival in Kidney Transplant Recipients
Source: Transpl Int. 2024 Aug 7;37:12574. doi: 10.3389/ti.2024.12574 (PMC11336573; doi:10.3389/ti.2024.12574)
Supplement: Supplementary file 1 [file DataSheet1.PDF]

**SUPPLEMENTARY MATERIALS**

**TABLE S1.** Baseline characteristics of participants according to systolic blood pressure categories in KOTRY.

**TABLE S2.** Outcome event rates according to baseline SBP in KOTRY.

**TABLE S3.** The hazard ratios for the composite outcome of CKD progression or graft failure according to baseline SBP or time-varying SBP in KOTRY.

**TABLE S4.** The hazard ratios for the composite outcome of CKD progression or graft failure according to SBP trajectory Patterns in KOTRY.

**List of KOTRY study group**

**Capsule Sentence Summary**

**TABLE S1. Baseline characteristics of participants according to systolic blood pressure categories in KOTRY**

| | SBP category (mmHg) | | | | | | |
|---|---|---|---|---|---|---|---|
| | **Total** | **<110** | **110-119** | **120-129** | **130-139** | **≥140** | **p-value** |
| **Demographic data** | | | | | | | |
| N (%) | 1,294 | 156 (12.1) | 241 (18.6) | 402 (31.1) | 308 (23.8) | 187 (14.5) | |
| Age (years) | 47.8 ± 11.4 | 47.0 ± 11.3 | 47.1 ± 11.1 | 47.7 ± 11.3 | 47.9 ± 12.1 | 49.0 ± 10.6 | 0.430 |
| Female, n (%) | 571 (44.1) | 82 (52.6) | 123 (51.0) | 167 (41.5) | 130 (42.2) | 69 (36.9) | 0.005 |
| BMI (kg/m2) | 23.0 ± 3.6 | 21.8 ± 3.4 | 22.4 ± 3.3 | 22.9 ± 3.6 | 23.5 ± 3.6 | 24.2 ± 3.5 | <0.001 |
| SBP (mmHg) | 125.4 ± 14.0 | 102.1 ± 6.0 | 114.7 ± 3.2 | 124.0 ± 3.1 | 133.7 ± 2.8 | 147.8 ± 7.8 | <0.001 |
| DBP (mmHg) | 77.0 ± 11.0 | 63.6 ± 7.6 | 71.5 ± 7.1 | 77.1 ± 8.5 | 81.6 ± 9.2 | 87.2 ± 10.1 | <0.001 |
| Diabetes mellitus, n (%) | 468 (36.2) | 58 (37.2) | 83 (34.4) | 129 (32.1) | 113 (36.7) | 85 (45.5) | 0.036 |
| Hypertension, n (%) | 973 (75.2) | 93 (59.6) | 162 (67.2) | 304 (75.6) | 249 (80.8) | 165 (88.2) | <0.001 |
| Smoker, n (%) | | | | | | | 0.990 |
| Never | 968 (74.8) | 118 (75.6) | 186 (77.2) | 301 (74.9) | 226 (73.4) | 137 (73.3) | |
| Current | 108 (8.3) | 13 (8.3) | 17 (7.1) | 34 (8.5) | 27 (8.8) | 17 (9.1) | |
| Former | 218 (16.8) | 25 (16.0) | 38 (15.8) | 67 (16.7) | 55 (17.9) | 33 (17.6) | |
| Donor, n (%) | | | | | | | 0.049 |
| Living donor | 907 (70.1) | 110 (70.5) | 179 (74.3) | 290 (72.1) | 213 (69.2) | 115 (61.5) | |
| Deceased or DCD | 387 (29.9) | 46 (29.5) | 62 (25.7) | 112 (27.9) | 95 (30.8) | 72 (38.5) | |
| Donor age (years) | 45.5 ± 12.9 | 43.8 ± 13.0 | 44.5 ± 12.9 | 45.1 ± 12.6 | 46.9 ± 13.0 | 46.6 ± 12.8 | 0.045 |
| Donor BMI (kg/m2) | 23.8 ± 3.3 | 23.5 ± 3.2 | 24.0 ± 3.4 | 24.1 ± 3.2 | 23.7 ± 3.5 | 23.6 ± 3.0 | 0.270 |
| Donor hypertension, n (%) | 162 (12.5) | 13 (8.3) | 24 (10.0) | 50 (12.4) | 44 (14.3) | 31 (16.6) | 0.110 |
| ABO-incompatibility, n (%) | 212 (16.4) | 27 (17.3) | 39 (16.2) | 64 (15.9) | 42 (13.6) | 40 (21.4) | 0.260 |
| Delayed graft function, n (%) | 47 (3.6) | 4 (2.6) | 9 (3.7) | 13 (3.2) | 10 (3.2) | 11 (5.9) | 0.470 |
| **Laboratory parameters** | | | | | | | |
| eGFR (ml/min/1.73m$^2$) | 64.5 ± 18.3 | 68.0 ± 19.2 | 66.0 ± 19.0 | 65.1 ± 18.6 | 63.3 ± 15.8 | 60.1 ± 19.1 | <0.001 |

| | | | | | | | |
|---|---|---|---|---|---|---|---|
| Donor eGFR (ml/min/1.73m$^2$) | 94.6 ± 73.0 | 107.7 ± 129.7 | 91.0 ± 34.1 | 95.0 ± 87.6 | 95.8 ± 42.7 | 85.7 ± 41.1 | 0.074 |
| Hemoglobin (g/dL) | 13.4 ± 1.9 | 13.0 ± 2.0 | 13.4 ± 1.9 | 13.6 ± 1.7 | 13.5 ± 1.8 | 13.4 ± 2.0 | 0.011 |
| Albumin (g/dL) | 4.3 ± 0.3 | 4.2 ± 0.4 | 4.2 ± 0.3 | 4.3 ± 0.3 | 4.3 ± 0.3 | 4.2 ± 0.4 | <0.001 |
| Fasting glucose (mg/dL) | 111.2 ± 36.8 | 108.2 ± 29.8 | 109.9 ± 32.8 | 110.2 ± 35.3 | 112.1 ± 43.0 | 116.5 ± 38.7 | 0.260 |
| T-Chol (mg/dL) | 176.6 ± 35.7 | 172.3 ± 35.4 | 177.1 ± 34.4 | 176.7 ± 34.0 | 177.6 ± 36.2 | 177.5 ± 40.2 | 0.620 |
| LDL-C (mg/dL) | 99.6 ± 30.8 | 98.7 ± 28.7 | 101.0 ± 30.3 | 97.5 ± 30.0 | 99.4 ± 30.1 | 103.3 ± 35.5 | 0.270 |
| HDL-C (mg/dL) | 58.7 ± 16.6 | 59.1 ± 16.5 | 57.9 ± 16.5 | 59.1 ± 16.8 | 60.1 ± 16.0 | 56.5 ± 17.1 | 0.180 |
| Triglyceride (mg/dL) | 135.2 ± 67.2 | 129.6 ± 62.4 | 132.4 ± 64.3 | 135.4 ± 71.7 | 133.1 ± 60.5 | 146.3 ± 74.1 | 0.140 |
| **Drugs** | | | | | | | |
| Tacrolimus, n (%) | 1,204 (93.0) | 144 (92.3) | 226 (93.8) | 377 (93.8) | 284 (92.2) | 173 (92.5) | 0.900 |
| Cyclosporine, n (%) | 75 (5.8) | 9 (5.8) | 13 (5.4) | 21 (5.2) | 20 (6.5) | 12 (6.4) | 0.950 |
| Steroid, n (%) | 1,200 (92.7) | 137 (87.8) | 224 (92.9) | 376 (93.5) | 285 (92.5) | 178 (95.2) | 0.100 |

Data are expressed as mean ± standard deviation, median [interquartile range], or proportion n (%).
Abbreviations: KOTRY, the Korean Organ Transplantation Registry; SBP, systolic blood pressure; BMI, body mass index; DBP, diastolic blood pressure; DCD, donation after circulatory death; eGFR, estimated glomerular filtration rate; T-chol, total cholesterol; LDL-C, low-density lipoprotein cholesterol; HDL-C, high-density lipoprotein cholesterol.

**TABLE S2. Outcome event rates according to baseline SBP in KOTRY**

| Outcomes | SBP categories (mmHg) | | | | | |
|---|---|---|---|---|---|---|
| | Overall | <110 | 110-119 | 120-129 | 130-139 | ≥140 |
| **No. of participants, n (%)** | 1294 | 156 (12.1) | 241 (18.6) | 402 (31.1) | 308 (23.8) | 187 (14.5) |
| **CKD progression**[a] | | | | | | |
| No. of person-years | 3382.4 | 425.1 | 621.2 | 1104.4 | 794.3 | 437.4 |
| Incidence of outcome, n (%) | 50 (3.9) | 5 (3.2) | 6 (2.5) | 16 (4.0) | 11 (3.6) | 12 (6.4) |
| Incidence rate per 1000 person-year | 14.8 | 11.8 | 9.7 | 14.5 | 13.8 | 27.4 |
| **Graft loss** | | | | | | |
| No. of person-years | 6061.3 | 739.9 | 1153.7 | 1922.7 | 1413.3 | 831.7 |
| Incidence of outcome, n (%) | 26 (2.0) | 4 (2.6) | 2 (0.8) | 6 (1.5) | 5 (1.6) | 9 (4.8) |
| Incidence rate per 1000 person-year | 4.3 | 5.4 | 1.7 | 3.1 | 3.5 | 10.8 |
| **Kidney composite outcome**[b] | | | | | | |
| No. of person-years | 3373.2 | 419.9 | 621.2 | 1103.2 | 793.4 | 435.5 |
| Incidence of outcome, n (%) | 58 (4.5) | 7 (4.5) | 6 (2.5) | 18 (4.5) | 13 (4.2) | 14 (7.5) |
| Incidence rate per 1000 person-year | 17.2 | 16.7 | 9.7 | 16.3 | 16.4 | 32.1 |

[a] CKD progression was defined as a decline of ≥ 50% in eGFR. [b] Composite outcome was defined as CKD progression or graft loss.
Abbreviations: KOTRY, the Korean Organ Transplantation Registry; CKD, chronic kidney disease; eGFR, estimated glomerular filtration rate, SBP, systolic blood pressure.

**TABLE S3. The hazard ratios for the composite outcome of CKD progression or graft failure according to baseline SBP or time-varying SBP in KOTRY**

| Baseline SBP | Model 1 | | Model 2 | |
|---|---|---|---|---|
| | HR (95% CI) | P value | HR (95% CI) | P value |
| <110 | 1.69 (0.57 - 5.04) | 0.344 | 1.67 (0.55 - 5.04) | 0.364 |
| 110-119 | 1.00 | | 1.00 | |
| 120-129 | 1.65 (0.66 - 4.16) | 0.287 | 2.06 (0.80 - 5.31) | 0.136 |
| 130-139 | 1.73 (0.66 - 4.55) | 0.267 | 2.10 (0.78 - 5.65) | 0.143 |
| ≥140 | 3.46 (1.33 - 9.01) | 0.011 | 3.85 (1.42 - 10.43) | 0.008 |
| **Time-varying SBP** | **Model 1** | | **Model 2** | |
| | HR (95% CI) | P value | HR (95% CI) | P value |
| <110 | 1.10 (0.39 - 3.08) | 0.863 | 1.61 (0.46 - 5.57) | 0.453 |
| 110-119 | 1.00 | | 1.00 | |
| 120-129 | 0.84 (0.36 - 1.94) | 0.677 | 1.42 (0.48 - 4.17) | 0.529 |
| 130-139 | 1.20 (0.53 - 2.75) | 0.663 | 1.61 (0.52 - 4.95) | 0.408 |
| ≥140 | 2.52 (1.09 - 5.82) | 0.031 | 4.16 (1.39 - 12.49) | 0.011 |

Model 1: Unadjusted. Model 2: Adjusted for age, sex, BMI, smoking status, DM, CVD, ABO compatibility, HLA compatibility, DGF, acute rejection during the first year, type of kidney donor (living or deceased donor), donor age, donor eGFR, donor BMI, donor hypertension, laboratory parameters (eGFR, hemoglobin, albumin, and LDL-C), and immunosuppressant use (tacrolimus, cyclosporine, and steroid).

Abbreviations: KOTRY (The Korean Organ Transplantation Registry); CKD, chronic kidney disease; SBP, systolic blood pressure; HR, hazard ratio; CI, confidence interval; BMI, body mass index; DM, diabetes mellitus; CVD, cardiovascular disease; HLA, human leukocyte antigen; DGF, delayed graft function; eGFR, estimated glomerular filtration rate; LDL-C, low-density lipoprotein cholesterol.

**TABLE S4. The hazard ratios for the composite outcome of CKD progression or graft failure according to SBP trajectory Patterns in KOTRY**

| | Model 1 | | Model 2 | |
|---|---|---|---|---|
| | **HR (95% CI)** | **P** | **HR (95% CI)** | **P** |
| Decreasing | 1.53 (0.68 - 3.43) | 0.304 | 0.69 (0.26 - 1.82) | 0.449 |
| Stable | 1.00 | | 1.00 | |
| Increasing | 1.52 (0.68 - 3.41) | 0.310 | 2.75 (1.10 - 6.84) | 0.030 |

Model 1: Unadjusted. Model 2: Adjusted for baseline SBP, age, sex, BMI, smoking status, DM, CVD, ABO compatibility, HLA compatibility, DGF, acute rejection during the first year, type of kidney donor (living or deceased donor), donor age, donor eGFR, donor BMI, donor hypertension, laboratory parameters (eGFR, hemoglobin, albumin, and LDL-C), and immunosuppressant use (tacrolimus, cyclosporine, and steroid).
Abbreviations: KOTRY (The Korean Organ Transplantation Registry); CKD, chronic kidney disease; SBP, systolic blood pressure; HR, hazard ratio; CI, confidence interval; BMI, body mass index; DM, diabetes mellitus; CVD, cardiovascular disease; HLA, human leukocyte antigen; DGF, delayed graft function; eGFR, estimated glomerular filtration rate; LDL-C, low-density lipoprotein cholesterol.

**KOTRY study group**

Myoung Soo Kim[1], Jaeseok Yang[2], Jin Min Kong[3], Ji Yoon Choi[4], Jun Young Lee[5], Cheol Woong Jung[6], Yeong Hoon Kim[7], Joong Kyung Kim[8], Chan-Duck Kim[9], Ji Won Min[10], Sung Kwang Park[11], Yeon Ho Park[12], Jae Berm Park[13], Jung Hwan Park[14], Jong-Won Park[15], Tae Hyun Ban[16], Sang Heon Song[17], Seung Hwan Song[18], Ho Sik Shin[19], Chul Woo Yang[20], Hye Eun Yoon[21], Kang Wook Lee[22], Dong Ryeol Lee[23], Dong Won Lee[24], Sam Yeol Lee[25], Sang-Ho Lee[26], Su Hyung Lee[27], Yu Ho Lee[28], Jung Pyo Lee[29], Jeong-Hoon Lee[30], Jin Seok Jeon[31], Heungman Jun[32], Kyunghwan Jeong[33], Ku Yong Chung[34], Hong Rae Cho[35], Ju Man Ki[36], Jong Cheol Jeong[37], Soo Jin Na Choi[38], Sung Shin[39], Seungyeup Han[40], Kyu Ha Huh[1]

[1]Department of Surgery, Yonsei University College of Medicine

[2]Department of Surgery, Seoul National University Hospital

[3]Department of Nephrology, BHS Hanseo Hospital

[4]Department of Surgery, College of Medicine, Han Yang University

[5]Department of Internal Medicine, Yonsei University Wonju College of Medicine, Wonju Severance Christian Hospital

[6]Department of Surgery, Korea University Anam Hospital

[7]Department of Internal Medicine, Inje University Busan Paik Hospital

[8]Department of Internal Medicine, Bongseng Memorial Hospital

[9]Department of Internal Medicine, School of Medicine, Kyungpook National University Hospital

[10]Division of Nephrology, Department of Internal Medicine, Bucheon St. Mary's Hospital

[11]Department of Internal Medicine, Jeonbuk National University Medical School

[12]Department of Surgery, Gil Medical Center, Gachon University College of Medicine

[13]Department of Surgery, Samsung Medical Center, Sungkyunkwan University School of Medicine

[14]Konkuk University School of Medicine, Department of Nephrology

[15]Department of Nephrology, Yeungnam University Hospital

[16]Division of Nephrology, Department of Internal Medicine, Eunpyeong St. Mary's hospital

[17]Department of Internal Medicine, Pusan National University Hospital

[18]Department of Surgery, Ewha Womans University Seoul Hospital

[19]Kosin University College of Medicine, Department of Internal Medicine, Division of Nephrology

[20]Division of Nephrology, Department of Internal Medicine, Seoul St. Mary's hospital

[21]Department of Internal Medicine, Incheon St. Mary’s Hospital, College of Medicine, The Catholic University of Korea College of Medicine

[22]Department of Nephrology, Chungnam National University Hospital

[23]Division of Nephrology, Department of Internal Medicine, Maryknoll Medical Center

[24]Division of Nephrology, Department of Internal Medicine, Pusan National University School of Medicine

[25]Department of Surgery, Kangdong Sacred Heart Hospital, Hallym University College of Medicine

[26]Department of Nephrology, Kyung Hee University Hospital at Gangdong

[27]Department of Surgery, Ajou University School of Medicine

[28]Division of Nephrology, Department of Internal Medicine, CHA Bundang Medical Center, CHA University, Seongnam, Korea

[29]Department of Nephrology, SMG-SNU Boramae Medical Center

[30]Department of Surgery, Myongji Hospital

[31]Department of Internal Medicine, Soonchunhyang University Seoul Hospital

[32]Department of Surgery, Inje University Ilsan Paik Hospital

[33]Department of Internal Medicine, Kyung Hee University College of Medicine

[34]Department of Surgery, Ewha Womans University Mokdong Hospital

[35]Department of Surgery, Ulsan University Hospital

[36]Department of Surgery, Gangnam Severance Hospital, Yonsei University College of Medicine

[37]Division of Nephrology, Seoul National University Bundang Hospital

[38]Department of Surgery, Chonnam National University Medical School

[39]Department of Surgery, Asan Medical Center

[40]Department of Internal Medicine, Keimyung University School of Medicine, Daegu, Korea

**Capsule Sentence Summary**

Systolic blood pressure ≥140 mmHg at 1 year after kidney transplantation and an increasing systolic blood pressure trajectory were associated with a higher risk of allograft dysfunction and failure in kidney transplant patients.
